# Transcriptome profile of carbon catabolite repression in an efficient l-(+)-lactic acid-producing bacterium *Enterococcus mundtii* QU25 grown in media with combinations of cellobiose, xylose, and glucose

**DOI:** 10.1371/journal.pone.0242070

**Published:** 2020-11-17

**Authors:** Yuh Shiwa, Haruko Fujiwara, Mao Numaguchi, Mohamed Ali Abdel-Rahman, Keisuke Nabeta, Yu Kanesaki, Yukihiro Tashiro, Takeshi Zendo, Naoto Tanaka, Nobuyuki Fujita, Hirofumi Yoshikawa, Kenji Sonomoto, Mariko Shimizu-Kadota

**Affiliations:** 1 Department of Molecular Microbiology, Tokyo University of Agriculture, Tokyo, Japan; 2 NODAI Genome Research Center, Tokyo University of Agriculture, Tokyo, Japan; 3 Division of Systems Bioengineering, Department of Bioscience and Biotechnology, Faculty of Agriculture, Graduate School, Kyushu University, Fukuoka, Japan; 4 Botany and Microbiology Department, Faculty of Science for Boys, Al-Azhar University, Cairo, Egypt; 5 Department of Bioscience, Tokyo University of Agriculture, Tokyo, Japan; 6 Department of Environmental Systems Sciences, Musashino University, Tokyo, Japan; University of Houston, UNITED STATES

## Abstract

*Enterococcus mundtii* QU25, a non-dairy lactic acid bacterium of the phylum Firmicutes, is capable of simultaneously fermenting cellobiose and xylose, and is described as a promising strain for the industrial production of optically pure l-lactic acid (≥ 99.9%) *via* homo-fermentation of lignocellulosic hydrolysates. Generally, Firmicutes bacteria show preferential consumption of sugar (usually glucose), termed carbon catabolite repression (CCR), while hampering the catabolism of other sugars. In our previous study, QU25 exhibited apparent CCR in a glucose-xylose mixture phenotypically, and transcriptional repression of the xylose operon encoding initial xylose metabolism genes, likely occurred in a CcpA-dependent manner. QU25 did not exhibit CCR phenotypically in a cellobiose-xylose mixture. The aim of the current study is to elucidate the transcriptional change associated with the simultaneous utilization of cellobiose and xylose. To this end, we performed RNA-seq analysis in the exponential growth phase of *E*. *mundtii* QU25 cells grown in glucose, cellobiose, and/or xylose as either sole or co-carbon sources. Our transcriptomic data showed that the xylose operon was weakly repressed in cells grown in a cellobiose-xylose mixture compared with that in cells grown in a glucose-xylose mixture. Furthermore, the gene expression of *talC*, the sole gene encoding transaldolase, is expected to be repressed by CcpA-mediated CCR. QU25 metabolized xylose without using transaldolase, which is necessary for homolactic fermentation from pentoses using the pentose-phosphate pathway. Hence, the metabolism of xylose in the presence of cellobiose by QU25 may have been due to 1) sufficient amounts of proteins encoded by the xylose operon genes for xylose metabolism despite of the slight repression of the operon, and 2) bypassing of the pentose-phosphate pathway without the TalC activity. Accordingly, we have determined the targets of genetic modification in QU25 to metabolize cellobiose, xylose and glucose simultaneously for application of the lactic fermentation from lignocellulosic hydrolysates.

## Introduction

Optically pure lactic acid is a feedstock for the production of poly-lactic acid (PLA), a biobased and biodegradable plastic [1], and of lactate-based polyesters containing 3-hydroxyalkanoates [2]. Bacterial fermentation from sustainable lignocellulosic biomass is a promising alternative source to fossil fuel-derived chemicals. After pretreatment and saccharification, the hydrolysates of the lignocellulosic biomass contain hexoses (predominant component, cellobiose, a β-1,4-linked glucose dimer) and pentoses (primarily xylose and arabinose) [3].

*Enterococcus mundtii* QU25 is a non-dairy lactic acid bacterium, isolated from ovine feces that can ferment both cellobiose and xylose to produce optically pure l-lactic acid (≥ 99.9%) *via* homo-fermentation [3–5]. Comparison of the results of lactic acid production from xylose shows that QU25 is one of the most efficient lactic acid-producing bacteria, giving a productivity of 6.15 (gL^-1^h^-1^), yield of 1.01 (gg^-1^), and final concentration of 41.0 (gL^-1^) [3,6,7]. In addition, QU25 utilizes cellobiose and xylose simultaneously for lactic fermentation [8]. Therefore, the industrial use of this strain has potential for facilitating the economical production of l-lactate from lignocellulosic biomass.

In general, Firmicutes bacteria show preferential consumption of specific sugars (generally glucose) due to transcriptional regulation, termed carbon catabolite repression (CCR), which hampers the catabolism of other sugars, such as xylose [9]. For example, catabolite control protein A (CcpA) is one of the most important, and highly conserved, transcriptional regulators of CCR in low-GC Firmicutes bacteria [10]. In the presence of a preferred sugar such as glucose, the cytosolic concentration of fructose 1,6-bisphosphate (FBP) increases through glycolysis. When the HPr kinase/phosphatase detects FBP, HPr becomes phosphorylated on residue Ser46 (HPr-Ser-P). Subsequently, the CcpA-(Hpr-Ser-P) complex binds cis-acting DNA motifs, called catabolite responsive elements (*cre*), thereby repressing the expression of genes or operons encoding metabolic enzymes for non-favorable sugars.

In our previous study, we reported that the QU25 strain exhibits apparent CCR in a glucose-xylose mixture, which is controlled at the transcriptional level with the repression occurring in initial xylose metabolism genes [11]. The QU25 genome encodes the main components of CCR such as CcpA, HPr, and HPr kinase/phosphatase [12]. Moreover, a putative *cre* has been identified upstream of the xylose operon. It is, therefore, plausible that the CcpA-(Hpr-Ser-P) complex serves as a repressor in the regulation of xylose utilization by QU25 in a glucose-xylose mixture [11]. Given that cellobiose is hydrolyzed into two glucose monomers by β-glucosidase and metabolized *via* FBP through glycolysis [13], cellobiose may trigger CcpA-dependent CCR in a cellobiose-xylose mixture. However, QU25 consumed xylose in the presence of cellobiose [8].

To elucidate the biological phenomenon associated with the simultaneous utilization of cellobiose and xylose, not that of glucose and xylose, in *E*. *mundtii* QU25, we performed RNA-seq analysis at the exponential growth phase for cells grown in glucose, cellobiose, and/or xylose as either their sole, or co-carbon sources. Our data demonstrated that in cellobiose-xylose grown cells, the transcriptional repression was still present in the xylose operon, but it relieved compared with that in the cells grown in a glucose-xylose mixture. Furthermore, QU25 could metabolize xylose without using transaldolase, which is a key enzyme of the pentose-phosphate (PP) pathway.

## Materials and methods

### Bacterial strain and media

In this study, the *Enterococcus mundtii* QU25 strain was used. A modified MRS medium (mMRS) was used for pre-culture and main culture [12]. As sugar sources, glucose, cellobiose, and xylose were used at a concentration of 70 g/L for single sugar cultivations, and 100 g/L glucose with 60 g/L xylose, or 100 g/L cellobiose with 60 g/L xylose for mixed sugar cultivations. In the pre-cultures, the same sugar sources as the main culture were used at one-tenth the concentration.

### Fermentation process

The cells were inoculated 1% (*v/v*) from a glycerol stock into 10 mL MRS medium and incubated overnight at 43°C to refresh cells. As a pre-culture, 4 mL of the refreshed cell cultures were inoculated in 36 mL mMRS medium and incubated at 43°C for 6 h. For the main culture, 40 mL of the pre-culture was transferred to a sterilized 1 L jar fermenter (Biott; Tokyo, Japan) containing 360 mL of mMRS medium. The pH was maintained at 7.0 using 10 M NaOH as a neutralizing agent, and the main culture was stirred at 100 rpm and cultured at 43°C. Samples (1.5 mL) for RNA-seq were collected from the main cultures 6 h after inoculation by centrifugation, and the obtained cells were frozen in liquid nitrogen and stored at ‒80°C until further use. Separately, samples (1 mL) were collected at different time intervals and analyzed for cell growth and composition of sugars and fermentation products, as described previously [8]. All cultures and samplings were performed in duplicate.

### RNA extraction, sequencing, and data analysis

Logarithmic phase cultures (6 h) were utilized for RNA extraction. Total RNA was extracted using RNAiso plus (TAKARA, Japan) according to the manufacturer’s instructions. The total RNA from each culture was prepared for analysis as follows: rRNA was extracted from each sample using the RiboZero Magnetic Kit for Gram-positive bacteria (Epicentre [an Illumina company], Madison, WI, USA). cDNA libraries were generated using the NEBNext mRNA Library Prep Reagent Set for Illumina (New England BioLabs, Ipswich, MA, USA).

Pooled cDNA libraries (overall 10, duplicates for five carbohydrates) were sequenced on an Illumina HiSeq2500 following the manufacturer’s protocols, which generated 100-bp paired-end reads and 6-bp index tags. Analyses of sequenced data were conducted using CLC Genomics Workbench (QIAGEN) ver. 11.0. The paired-end reads were subjected to quality trimming (removal of adapter sequences and low-quality sequences with a quality limit of 0.05, two ambiguous nucleotides allowed per read, and removal of 3' terminal nucleotides 50). Trimmed reads were mapped to the QU25 genome (NC_022878–NC_022884) using an RNA-seq tool with default parameters. Only chromosomal genes were retained for further analysis.

For visualization of global gene expression, hierarchical clustering using Euclidean distance and complete linkage based on differentially expressed genes (DEGs) among growth conditions were performed using the Create Heat Map tool for RNA-seq. DEGs among growth conditions were identified by one-way ANOVA using a threshold of fold change > 2 and false discovery rate (FDR) p-value < 0.05. For the statistical analysis of DEGs in a pairwise comparison, the Differential Expression tool for RNA-seq was used to produce a DEG table with data comparisons for all pairs. The genes were considered to be DEGs if they had a FDR p-value < 0.05 and fold change ≥ 2 for the comparisons. Operon structures were predicted using Genome 2D (http://genome2d.molgenrug.nl) [14]. We searched for *cre* in the QU25 genome using the query sequence (WTGNNARCGNWWWCAW, where W stands for A or T, R for A or G, and N for any base) [15]. CRE sequences were identified using *in silico* Molecular Cloning Genomics Edition software (In Silico Biology, Inc., Yokohama, Japan) [16]. The Kyoto Encyclopedia of Genes and Genomes (www.genome.jp/kegg/) database was used to analyze orthologs.

### RNA-seq data accession numbers

The RNA-seq data obtained in this study were deposited in the DNA Data Bank of Japan Sequence Read Archive (DRA)/National Center for Biotechnology Information Sequence Read Archive (SRA)/European Bioinformatics Institute Sequence Read Archive (ERA) under the accession number DRA009597.

## Results

### Fermentation profiles of QU25 grown with single or mixed sugars

To examine the transcriptome of strain QU25 under various sugar conditions, we performed RNA-seq analysis on QU25 cultured in glucose, cellobiose, xylose, a glucose-xylose mixture, and a cellobiose-xylose mixture at the exponential growth phase (S1 Fig). Statistical data for the transcriptomes, including the number of reads per sample, and mapping ratios to reference are summarized in S1 Table.

Fermentation profiles under the sugar conditions are shown in S1 Fig. As previously shown [8], QU25 grown in the glucose-xylose mixture exhibited apparent CCR of xylose utilization, while QU25 grown in the cellobiose-xylose mixture exhibited simultaneous consumption of both cellobiose and xylose (S1 Fig).

### Differences in transcriptome data profiles under various sugar conditions

**Fig 1:**
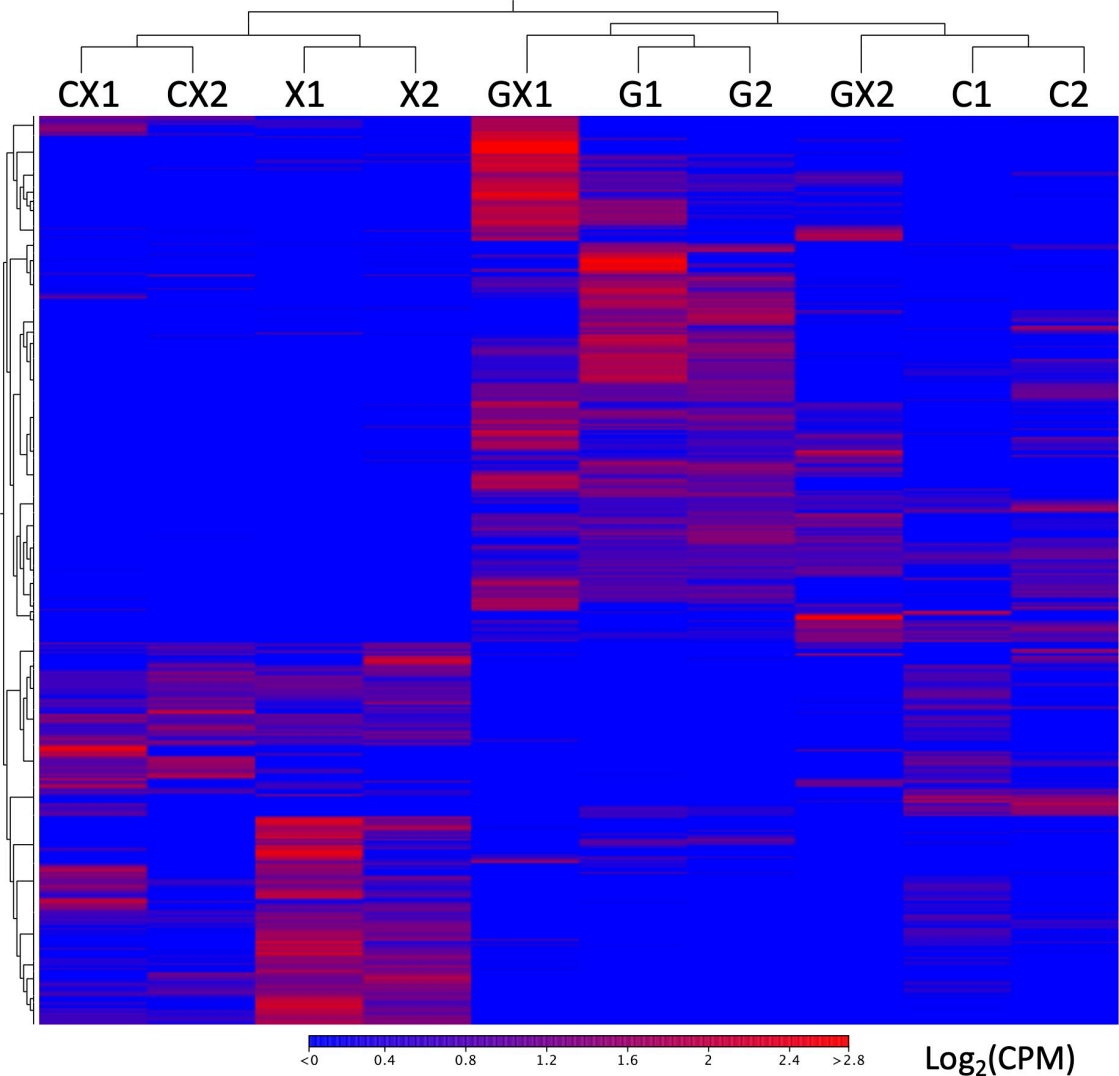
Heat map and hierarchical clustering of genes and samples for 816 differentially expressed genes. DEGs were identified by one-way ANOVA (> 2-fold change, FDR < 0.05). Abbreviations: *G*, glucose; *X*, xylose; *C*, cellobiose; *GX*, glucose and xylose mixture; *CX*, cellobiose and xylose mixture. Different replicates for each carbon source are numbered (e.g., G1, G2).

To assess the levels of difference between transcriptomes, we performed hierarchical clustering of the samples based on DEGs identified among growth conditions (> 2-fold change, FDR < 0.05, n = 816; Fig 1). For single sugar cultivations, the glucose samples clustered together with the cellobiose samples, while the xylose samples formed a clear district cluster. Interestingly, cellobiose induced more than 50 genes that were not induced by glucose at the transcriptional level (see the C/G columns in Table 1), while the cellobiose samples clustered separately from the glucose samples. For transcriptomes of the mixed sugar cultivations, the samples of the glucose-xylose mixture clustered together with the glucose and cellobiose samples, whereas the samples of the cellobiose-xylose mixture clustered together with the xylose samples. In addition, a significant number of genes induced by xylose were still transcribed and not repressed in the cellobiose-xylose mixture at the transcriptional level. These results indicate that gene expression patterns in cells grown in the cellobiose-xylose mixture were distinct from those of the glucose-xylose mixture, even though cellobiose is composed of a glucose dimer.

**Table 1:** Number of genes expressed differentially between two growth conditions.

Pairwise comparisons	C/G	X/G	X/C	GX/X	CX/C	CX/X	CX/GX
No. of upregulated genes	55	266	74	231	25	23	174
No. of downregulated genes	88	235	100	232	69	54	202
No. of total genes	143	501	174	463	94	77	376

Abbreviations: *G*, glucose; *X*, xylose; *C*, cellobiose; *GX*, glucose and xylose mixture; *CX*, cellobiose and xylose mixture. DEGs were identified as genes with an FDR p-value < 0.05 and fold change ≥ 2. C/G indicates the comparison between cellobiose and glucose transcriptomes, and upregulation and downregulation were determined through the comparison between the cellobiose transcriptome and the glucose transcriptome.

Differential expression analysis was performed as pairwise comparisons among five different transcriptomes. Total transcriptome data for all growth conditions can be found in S2 Table. The total number of DEGs reflected the detected relationships between the different transcriptomes (Table 1). The number of DEGs between the samples of the cellobiose-xylose mixture and the xylose samples (CX/X in Table 1) was approximately one-sixth of that between the samples of the glucose-xylose mixture and xylose samples (GX/X in Table 1), indicating that the gene expression pattern of cells grown in the cellobiose-xylose mixture was more similar to that in the xylose medium compared to that in the glucose-xylose mixture, and that the results of this analysis are consistent with those of clustering shown in Fig 1.

### Genes involved in cellobiose utilization

Cellobiose is generally transported across the cell membrane of Firmicutes by the phosphoenolpyruvate: carbohydrate phosphotransferase system (PTS) and hydrolyzed by β-glucosidase [13]. Analysis of differentially upregulated loci in the cellobiose samples compared to those in the glucose samples was used to identify genes related to cellobiose uptake and hydrolysis in QU25, since more than 70 PTS and 10 glucosidase genes were found in the QU25 genome. Genes that were found to be significantly upregulated are listed in S3 Table. Cellobiose induced genes for five putative PTS genes/clusters (EMQU_0307–0309, EMQU_0875–0877, EMQU_1036, EMQU_2186, and EMQU_2681). In addition, the genes for three glycoside hydrolase family 1 proteins (EMQU_0608, EMQU_1037, and EMQU_2185), which were annotated as 6-phospho-beta-glucosidase in KEGG, were significantly upregulated in the cellobiose samples compared to the glucose samples. These data showed that QU25 contained multiple cellobiose-inducible genes responsible for cellobiose uptake via PTS and metabolism. Further, the presence of xylose was not likely to affect the transcript amounts of the PTSs and β-glucosidase genes as these genes were not significantly differentially expressed in the cells grown in the cellobiose-xylose mixture compared to those of the cellobiose medium (see the CX/C columns in S3 Table).

### Genes involved in initial xylose metabolism

Our previous study showed that genes involved in initial xylose utilization (nonPTS transporter, xylose isomerase, and xylulokinase) formed the xylose operon (Table 2) [11,12]. After incorporation of xylose into QU25 cells, xylose is converted to the common metabolic intermediate xylulose 5-phosphate by xylose isomerase and xylulokinase, products of the genes in the xylose operon. In QU25 grown at high xylose concentrations, our previous genome and transcriptome analyses predicted that xylulose 5-phosphate is converted to lactic acid as a final product *via* the homofermentative pentose-phosphate/glycolytic pathway [11,12]. In the opposite direction to the xylose operon, *xylR* (EMQU_2811) is present, which is a ROK family transcriptional repressor of the xylose operon. The putative XylR-binding site and *cre* have been identified upstream of this xylose operon, indicating that XylR and CcpA are likely involved in the regulation of this xylose operon [11].

**Table 2:** Statistically upregulated or downregulated genes involved in xylose catabolism.

Gene ID	Gene name	Gene Product (NCBI RefSeq)	Mean RPKM					Fold change			
			G	C	X	GX	CX	X/G	GX/X	CX/X	CX/GX
Initial xylose utilization genes											
EMQU_2811	*xylR*	ROK family transcriptional regulator	36.0	23.6	117.4	335.4	148.6	**3.26**	2.86	1.27	0.44
EMQU_2810	*xylA*	xylose isomerase	3.6	2.5	2880.7	35.7	453.9	**790.43**	**0.01**	0.16	**12.70**
EMQU_2809	*xynB*	glycoside hydrolase family 43 protein	2.7	1.5	2510.4	23.5	364.3	**940.83**	**0.01**	0.15	**15.49**
EMQU_2808	*araG*	sugar ABC transporter ATP-binding protein	4.5	1.7	1046.5	9.4	149.4	**231.17**	**0.01**	**0.14**	**15.96**
EMQU_2805	*xylB*	xylulokinase	16.0	10.5	972.6	10.5	138.5	**60.81**	**0.01**	**0.14**	**13.20**
Pentose-phosphate pathway											
EMQU_1275	*tktA1*	transketolase	990.7	340.9	451.9	214.4	446.7	0.46	0.47	0.99	2.08
EMQU_2812	*tktA3*	transketolase	4.5	3.8	152.2	9.7	12.5	**33.52**	**0.06**	**0.08**	1.28
EMQU_2814	*talC*	fructose-6-phosphate aldolase	1.5	1.8	266.5	2.2	5.3	**175.94**	**0.01**	**0.02**	2.41
Bypass of pentose-phosphate pathway											
EMQU_1003	*pfk*	6-phosphofructokinase	422.3	287.1	217.2	252.5	393.5	0.51	1.16	1.81	1.56
EMQU_1665	*pfk*	6-phosphofructokinase	7.7	8.3	25.8	2.2	16.1	3.34	**0.09**	0.62	**7.27**
EMQU_0715	*fba*	fructose-bisphosphate aldolase	1247.5	1981.4	1267.1	1458.1	2105.6	1.02	1.15	1.66	1.44
CCR proteins											
EMQU_0954	*ptsH (HPr)*	phosphocarrier protein HPr	976.8	2275.5	2547.8	646.0	3230.7	2.61	**0.25**	1.27	**5.00**
EMQU_1943	*ccpA*	catabolite control protein A	91.6	148.0	132.3	267.6	226.3	1.44	2.02	1.71	0.85
EMQU_1951	*hptK*	HPr kinase/phosphorylase	93.3	125.2	114.4	127.9	103.2	1.23	1.12	0.90	0.81

For designations, such as X/G, see Table 1. Statistically significant fold change values are shown in bold (FDR p-value < 0.05 and fold change ≥ ±2).

We then examined the expression of genes involved in xylose metabolism (Table 2). The expression of the xylose operon in cells grown in the medium only containing xylose or glucose suggested that the operon was transcribed by xylose and repressed by glucose. Further, the predicted XylR-binding site was likely to be functional (Table 2, column G and X). The expression levels of the initial xylose metabolism genes were approximately 100-fold lower in the glucose-xylose mixture samples than in the xylose samples (Table 2, column GX/X), indicating that glucose-mediated transcriptional repression occurred, likely due to the *cre*/CcpA complex. These data are consistent with our previous results, which demonstrate a gradual reduction in the expression of several genes in the xylose operon with the increase of the glucose concentration in the glucose-xylose mixture [11].

In spite of the simultaneous consumption of cellobiose and xylose, the transcriptional level of the initial xylose metabolism genes was approximately 7-fold lower in the cellobiose-xylose mixture than that in the xylose samples (Table 2, column CX/X). Meanwhile, the expression levels of the initial xylose metabolism genes were approximately 15-fold higher in samples of the cellobiose-xylose mixture than those in the samples of the glucose-xylose mixture (Table 2, column CX/GX). Taken together, these results indicate that, in cellobiose-xylose grown cells, transcriptional repression of the xylose operon occurs but at a lower level than that observed in QU25 cells grown in the glucose-xylose mixture.

### Genes involved in CcpA-dependent CCR

There are several key proteins encoded by the QU25 genome that likely participate in the CcpA-dependent CCR pathway of Firmicutes in response to glucose, including CcpA (EMQU_1943), histidine-containing protein (HPr) (EMQU_0954), and HPr kinase/phosphorylase (EMQU_1951) [11,12]. While *ccpA* and *hprK* (encoding HPr kinase/phosphorylase) were constitutively expressed under all conditions tested, the expression of *ptsH* (HPr) was approximately one-fourth times lower in the samples of the glucose-xylose mixture compared to that in the xylose samples (Table 2, column GX/X), and five-times higher in the samples of the cellobiose-xylose mixture compared to those of the glucose-xylose mixture (Table 2, column CX/GX).

### Genes in the pentose-phosphate (PP) pathway

Transketolase and transaldolase are key enzymes in the PP pathway, and support homolactic fermentation from pentoses in QU25 [4]. In the QU25 genome, two transketolase genes and one transaldolase gene were identified. As for the transketolase genes, EMQU_1275 (tktA1) was constitutively expressed under all conditions tested, whereas EMQU_2812 (tktA3) was highly induced only in the xylose samples. The amount of the transketolase transcript in the samples grown in the cellobiose-xylose mixture was likely sufficient for transketolase enzymatic activity, which supports xylose metabolism.

The only annotated gene that encodes transaldolase, *talC* (EMQU_2814), which degrades sedoheptulose 7-phosphate (S7P), was highly induced by xylose; while very low amounts of the *talC* transcript were detected in the cells grown in glucose, cellobiose, glucose-xylose, and cellobiose-xylose media compared to those in xylose (Table 2). A putative *cre* (TTGACAGCGATTTCAT; perfect match to the *cre* consensus sequence) sequence was identified at position -1078 upstream of *talC* (EMQU_2814) start codon, and was located in the coding region of an adjacent gene (EMQU_2815) predicted within the same operon, suggesting that *talC* expression in QU25 is likely repressed by CcpA-mediated CCR. In the cellobiose-xylose mixture, the amount of the *talC* transcript appeared to be insufficient for transaldolase enzymatic activity.

Nakahigashi et al. reported bypass of the PP in transaldolase-deficient mutants of *Escherichia coli* to allow for the metabolism of xylose [17]. In the bypass pathway, S7P was converted to erythrose 4-phosphate and dihydroxyacetone phosphate *via* S1,7P by the universally conserved glycolytic enzymes, ATP-dependent phosphofructokinase (*pfk*; 6-phosphofructokinase) and aldolase (*fba*; fructose-bisphosphate aldolase). Genes encoding both of these enzymes (*pfk*, EMQU_1003 and EMQU_1665; and *fba*, EMQU_0715) were highly expressed in the cellobiose-xylose mixture of QU25 cells (Table 2). It is likely that this PP bypass pathway for S7P degradation is functional in the cells grown in the cellobiose-xylose mixture and responsible for xylose metabolism, similar to *tal*-deficient mutants of *E*. *coli*.

## Discussion

Though lignocellulosic biomasses are one of the most abundant and sustainable resources, the development of methods to enable their utilization for biofuels and biobased materials has been slow. Since lignocellulosic hydrolysates contain both hexoses and pentoses, CCR is one of the difficulties hindering their use as substrates for microbial fermentations to produce both biobased materials and biofuels. Many attempts have been made to resolve this issue. For instance, in ethanologenic *Escherichia coli*, the gene for a CCR target protein, glucose phosphotransferase (*ptsG*), was mutated to achieve simultaneous consumption of glucose and pentoses to produce ethanol [18]. Furthermore, to facilitate ethanol production, engineered strains of eukaryotic yeast carrying exogenous genes have successfully evaded CCR, as newly introduced genes for the transporters and metabolic enzymes do not contain target domains for the repression machinery [19]. However, considering that heterogenetic enzymes may produce inhibitors for sugar metabolism in a new environment, careful experimental design is necessary [20].

Optically pure lactic acid is a biobased feedstock that is generally produced by homolactic fermentation with lactic acid bacteria from not only hexoses but also pentoses via the PP pathway. Among the facultative anaerobic Firmicutes, non-dairy enterococci have recently become a primary focus for application in the industrial production of lactic acid in America, Europe, Asia and Africa [21–25]. Some of these bacteria are capable of growing at relatively high temperatures (> 45°C) [26], which reduces contamination and energy requirements for cooling, and facilitates the adoption of simultaneous saccharification and fermentation (SSF) [3].

*E*. *mundtii* QU25 has been suggested as a candidate organism for the fermentation of lignocellulose due to its superior production of lactic acid and its ability to consume xylose in the presence of cellobiose during lactic fermentation. Though the most effective temperature for lactic acid production of QU25 was 43°C, this strain is able to grow at 50°C [5] and has potential for SSF. QU25 exhibits apparent transcriptional CCR on glucose and xylose sugar mixtures and its genome encodes the complete CcpA-dependent CCR system typical for Firmicutes bacteria. To understand the simultaneous consumption of cellobiose and xylose in QU25, we analyzed transcriptional profiles in QU25 cultures under various sugar conditions at the exponential growth phase.

The gene expression pattern in the whole genome of QU25 cells grown in the cellobiose-xylose mixture was more similar macroscopically to that of cells grown in xylose compared to that of cells grown in the glucose-xylose mixture (Fig 1). Moreover, a significant number of xylose-induced genes were not repressed and still transcribed in cells grown in the cellobiose-xylose mixture.

Focusing on the xylose operon genes responsible for the incorporation and initial xylose metabolism including genes encoding xylose isomerase and xylulokinase, we demonstrated that the operon genes were still repressed in the cells grown in the cellobiose-xylose mixture compared to those in the xylose medium. However, the amounts of their transcripts were more compared to those in the glucose-xylose mixture (Table 2, columns CX/X and CX/GX). These results are consistent with our previous results which demonstrated that the enzymatic activities of xylose isomerase and xylulokinase were three-fold higher in cells grown in a cellobiose-xylose mixture than those in cells grown in a glucose-xylose mixture [8]. We, therefore, postulate that the amounts of transcripts in cells grown in the cellobiose-xylose mixture were sufficient to facilitate xylose metabolism. Furthermore, the presence of xylose did not affect the amounts of transcripts generated for cellobiose metabolism in the samples of the cellobiose-xylose mixture (S3 Table), which agrees with our results indicating significantly higher beta-glucosidase activity in cells grown in a cellobiose-xylose mixture than those in a glucose-xylose mixture [8].

Furthermore, our transcriptomic data shows that QU25 effectively metabolized xylose under low transcription of *talC* (Table 2), suggesting that QU25 uses the bypass pathway for S7P degradation in the PP pathway when grown in the cellobiose-xylose mixture. This bypass pathway was found by Nakahigashi *et al* and may be commonly employed by various microorganisms [17]. In addition, using LC/MS/MS, we qualitatively detected S1,7P, an intermediate of the bypass pathway, after preliminarily adding of xylulose 5-phosphate and ribulose 5-phosphate to cell lysates of the QU25 grown in xylose (S2 Fig). These results further support the possibility that QU25 bypasses the PP pathway for S7P degradation in xylose metabolism.

In our previous study, the CcpA-(Hpr-Ser-P) complex acting as a general repressor for CCR had been supposed to be relieved in cells grown in cellobiose and xylose mixture [8], however, under these conditions, the transcription of *talC*, which has a perfect *cre* sequence upstream of the initiation codon, was tightly repressed (see Table 2). Therefore, we have examined other possibilities for the reduction of transcriptional repression specific for xylose operon genes in the cellobiose-xylose mixture.

Transcriptional regulators, including PTS regulation domains (PRDs) and sometimes EIIA and EIIB domains, were previously reported as being involved in the CCR mechanisms [27]. In *Clostridium acetobutylicum*, it has been reported that the PRDs-containing transcriptional activator CelR and sigma factor σ54 (*sigL* product) regulated cellobiose utilization [28]. We identified orthologs of two *celR* genes (EMQU_0827 and EMQU_0874), ten PRD domain-containing genes (S2 Table), and one *sigL* (EMQU_2018) in the QU25 genome. In *C*. *acetobutylicum*, CelR binds to upstream activator sequences (UAS) at sites 80–150 bp upstream of the *sigL* promoter [28], and in QU25, putative cellobiose PTS genes (EMQU_0875–0877) with a *sigL* promoter located downstream of one of the *celR* orthologs (EMQU_0874), were highly induced by cellobiose (S3 Table). However, we were unable to identify UAS and *sigL* promoter sequences in the upstream regions of the xylose operon in QU25. It is, therefore, possible that unknown PRDs-containing cellobiose-responsive transcriptional activator(s) bind to the promoter region of the xylose operon and function in the upregulation of the xylose operon in the cells grown in the cellobiose-xylose mixture, in the presence of typical cre-CcpA-dependent CCR (S3 Fig).

Herein, we have successfully described the use of *E*. *mundtii* QU25, for the fermentation of lignocellulosic hydrolysates by the simultaneous consumption of various sugars, which can be readily applied to industrial level production of optically pure lactic acid. Further studies to advance this work will include the development of a system for the genetic modification of this strain, similar to that previously reported for the successful engineering of microorganisms to overcome CCR. Next, we will attempt to delete the *cre* sequence upstream of the xylose operon to avoid binding the CcpA complex to the sequence, which serves as a repressor. We will also add a binding sequence for the putative positive transcriptional regulator activated by cellobiose, the existence of which in QU25 was predicted in this study.

## Supporting information

S1 Fig **Fermentation profiles of lactic acid production in cellobiose-xylose (A), glucose-xylose (B), glucose (C), cellobiose (D), and xylose (E) sugar mixtures.** *E*. *mundtii* QU25 was cultured in a 1 L jar fermenter containing 360 mL mMRS medium at 43°C with 100 rpm agitation and at pH 7.0 (adjusted with 10 M NaOH). The sampling points for RNA-seq are indicated by arrows.
(TIFF)

S2 Fig *In vitro* detection of S7P and S1,7P, intermediates of the PP pathway. Amounts of S7P and S1,7P extracted from the enzymatic reaction mixture were measured by LC-MS/MS. The crude extracts for the enzymatic assays were obtained from cells grown in xylose. The reaction mixture (50 μL) was incubated with 25 μL of MOPS-KOH buffer (pH 7.2; 1 M), 2.5 μL of ribulose 5-phosphate (25 mM), 2.5 μL of xylulose 5-phosphate (25 mM), 2.5 μL of MgCl_2_ (100 mM), 2.5 μL of ATP (100 mM), and 2.5 μL of crude extracts at 37°C for 1 and 2 h. The filtered supernatant was analyzed by liquid chromatography-tandem mass spectrometry (LC-MS/MS) using a LCMS-8050 triple quadrupole mass spectrometer system (Shimadzu, Kyoto, Japan) with a reversed-phase Mastro SP column (2.1 × 100 mm; Shimadzu) using the same analytical conditions as previously described [29]. Quantification of S7P and S1,7P was performed with the standard curve obtained using various concentrations.
(TIF)

S3 Fig Schematic representation of two potential mechanisms to relieve transcriptional repression of the xylose gene operon in the cellobiose-xylose mixture sample. Abbreviations: IIA, IIB, IIC components of cellobiose-specific PTS; Cellobiose 6-P, cellobiose 6-phosphate; FBP, fructose 1,6-bisphosphate; CcpA, carbon catabolite protein A; HPr, histidine-containing protein; HPr-Ser-P, phosphorylated form of HPr; HPrK, HPr kinase/phosphatase; *cre*, catabolite responsive elements; PRD-TA; PRDs-containing transcriptional activators. This illustration was created using BioRender.com.
(TIF)

S1 Table Transcriptome statistical data, including the number of reads per sample and mapping ratio to the reference. (XLSX)

S2 Table Total transcriptome data representing all growth conditions from this study. The data table consists of gene locus tag ID, predicted operon ID using Genome 2D, gene annotation from NCBI RefSeq and KEGG, RPKM values (Reads Per Kilobase per Million reads sequenced), and fold change values with corresponding p-values. Statistically significant fold change values are shown in bold (FDR p-value < 0.05 and fold change ≥ 2). For the designations, such as X/G, see Table 1.
(XLSX)

S3 Table Statistically significant upregulated genes involved in cellobiose catabolism. For the designations, such as X/G, see Table 1. Statistically significant fold change values are shown in bold (FDR p-value < 0.05 and fold change ≥ 2).
(XLSX)
