# Supplementary figures and images for: Transcriptome profile of carbon catabolite repression in an efficient l-(+)-lactic acid-producing bacterium Enterococcus mundtii QU25 grown in media with combinations of cellobiose, xylose, and glucose
Source: PLoS One. 2020 Nov 17;15(11):e0242070. doi: 10.1371/journal.pone.0242070 (PMC7671544; doi:10.1371/journal.pone.0242070)

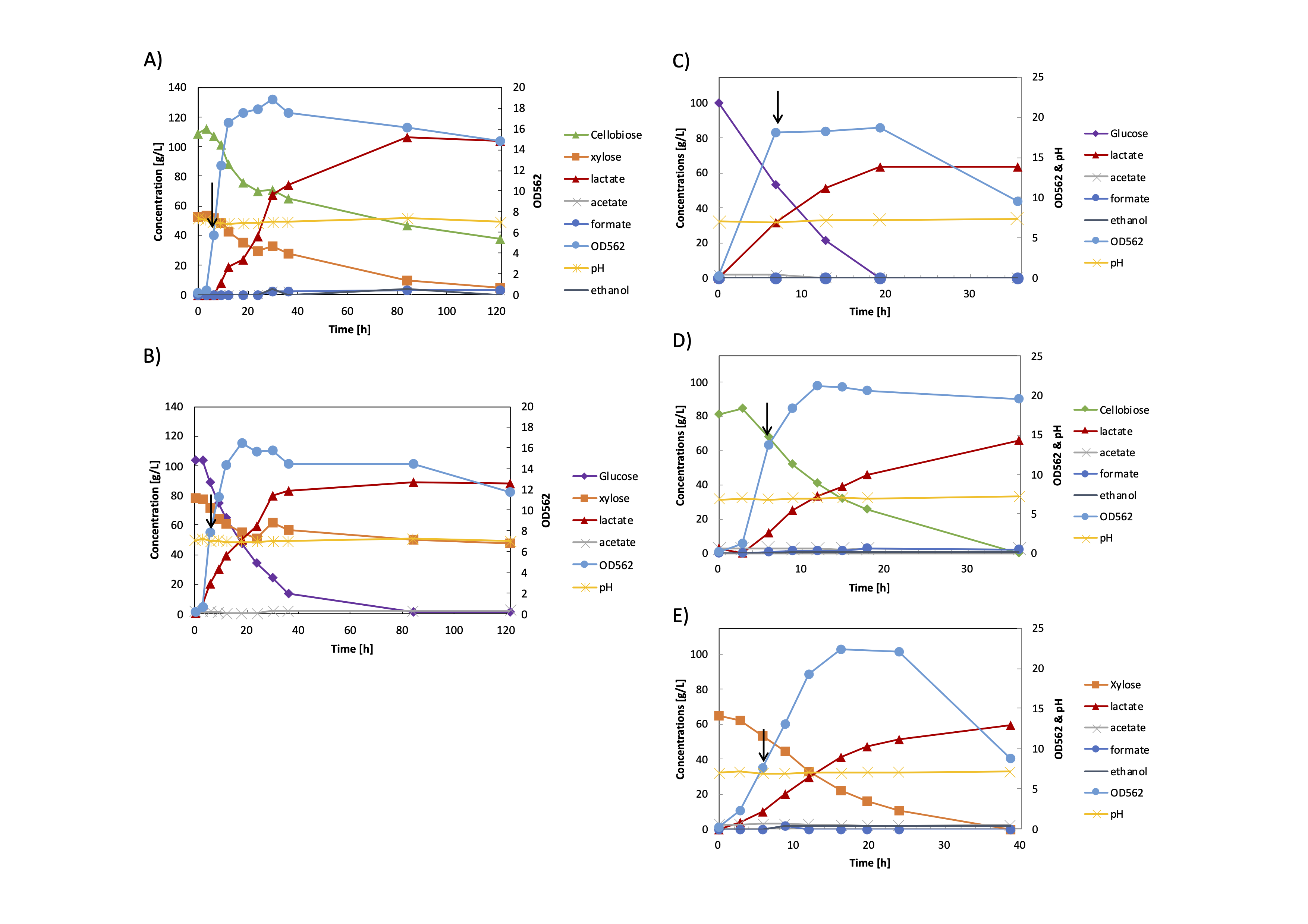

Supplement: S1 Fig — Fermentation profiles of lactic acid production in cellobiose-xylose (A), glucose-xylose (B), glucose (C), cellobiose (D), and xylose (E) sugar mixtures. E. mundtii QU25 was cultured in a 1 L jar fermenter containing 360 mL mMRS medium at 43°C with 100 rpm agitation and at pH 7.0 (adjusted with 10 M NaOH). The sampling points for RNA-seq are indicated by arrows. (TIFF) [file pone.0242070.s001.tiff]

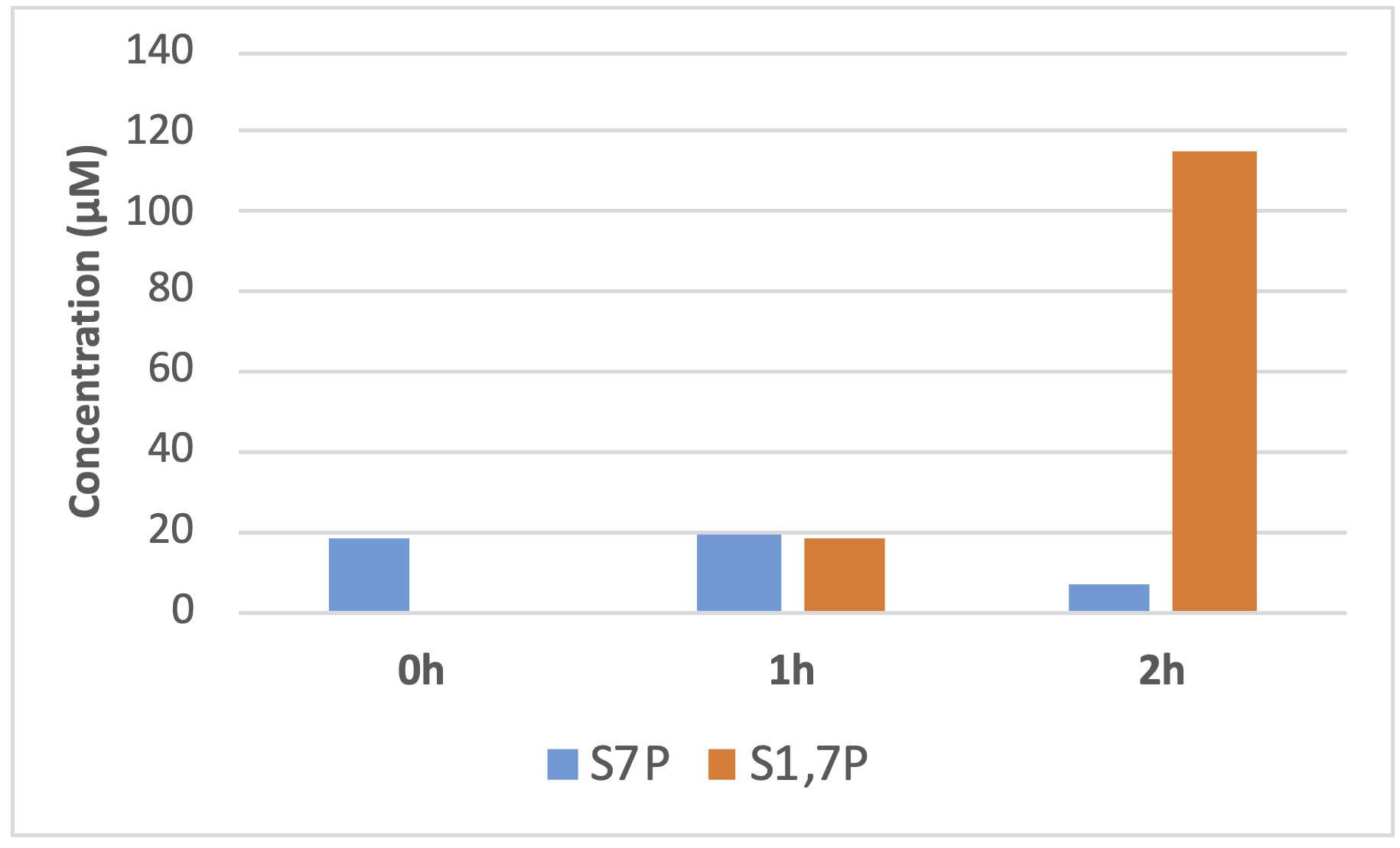

Supplement: S2 Fig — Amounts of S7P and S1,7P extracted from the enzymatic reaction mixture were measured by LC-MS/MS. The crude extracts for the enzymatic assays were obtained from cells grown in xylose. The reaction mixture (50 μL) was incubated with 25 μL of MOPS-KOH buffer (pH 7.2; 1 M), 2.5 μL of ribulose 5-phosphate (25 mM), 2.5 μL of xylulose 5-phosphate (25 mM), 2.5 μL of MgCl2 (100 mM), 2.5 μL of ATP (100 mM), and 2.5 μL of crude extracts at 37°C for 1 and 2 h. The filtered supernatant was analyzed by liquid chromatography-tandem mass spectrometry (LC-MS/MS) using a LCMS-8050 triple quadrupole mass spectrometer system (Shimadzu, Kyoto, Japan) with a reversed-phase Mastro SP column (2.1 × 100 mm; Shimadzu) using the same analytical conditions as previously described [29]. Quantification of S7P and S1,7P was performed with the standard curve obtained using various concentrations. (TIF) [file pone.0242070.s002.tif]

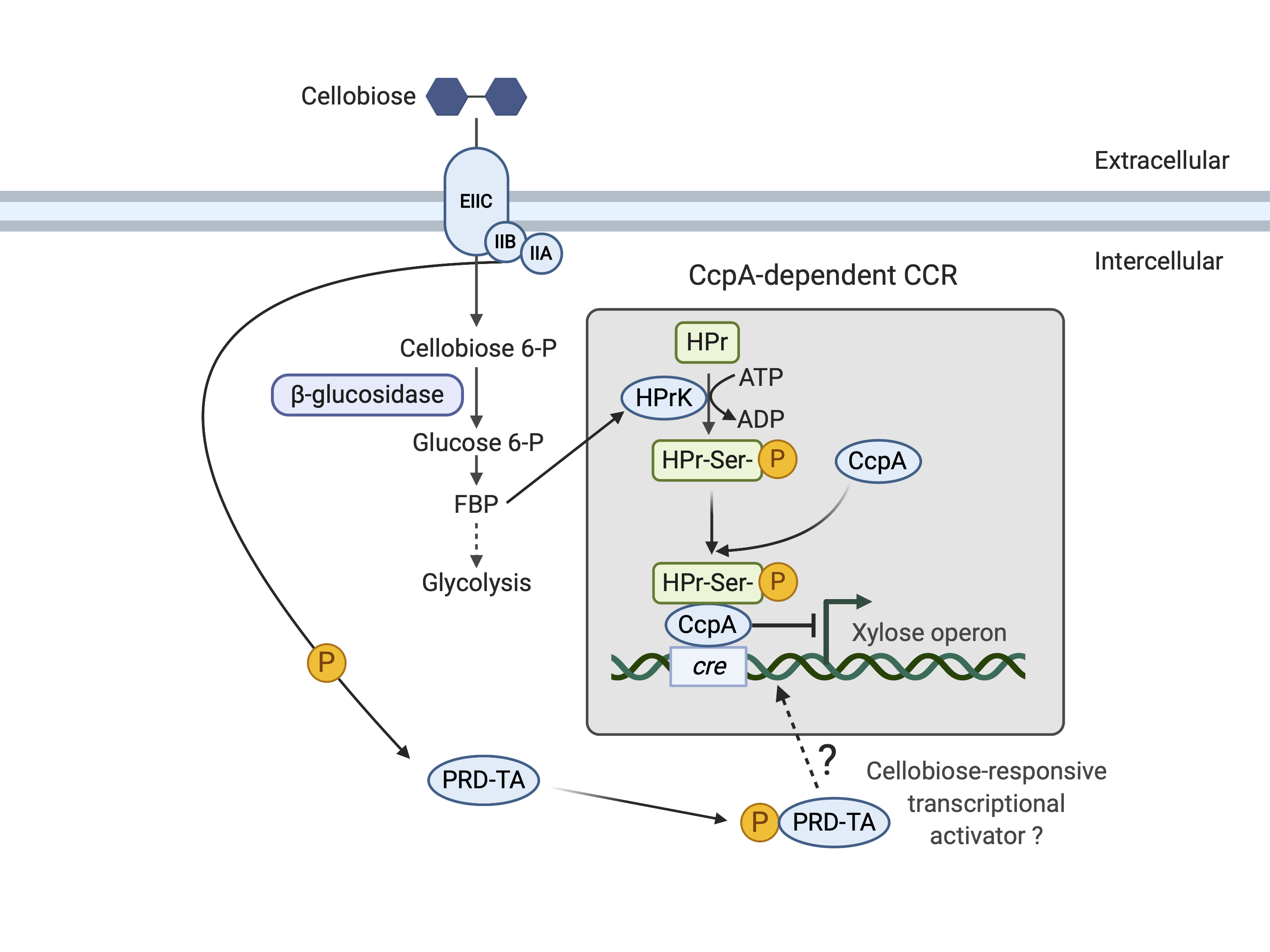

Supplement: S3 Fig — Abbreviations: IIA, IIB, IIC components of cellobiose-specific PTS; Cellobiose 6-P, cellobiose 6-phosphate; FBP, fructose 1,6-bisphosphate; CcpA, carbon catabolite protein A; HPr, histidine-containing protein; HPr-Ser-P, phosphorylated form of HPr; HPrK, HPr kinase/phosphatase; cre, catabolite responsive elements; PRD-TA; PRDs-containing transcriptional activators. This illustration was created using BioRender.com. (TIF) [file pone.0242070.s003.tif]
